# Patient-provider communication while using a clinical decision support tool: explaining satisfaction with shared decision making for mammography screening

**DOI:** 10.1186/s12911-022-02058-3

**Published:** 2022-12-07

**Authors:** Yan Liu, Rachel Kornfield, Ellie Fan Yang, Elizabeth Burnside, Jon Keevil, Dhavan V. Shah

**Affiliations:** 1 School of Journalism and Communication, Shanghai University, 149 Yanchang Drive, 1006 Xingjian Building, Shanghai, 200072 China; 2 Department of Preventive Medicine, Northwestern University, Chicago, IL 60611 USA; 3 School of Communication and Mass Media, Northwest Missouri State University, Maryville, MO 64468 USA; 4 School of Medicine and Public Health, University of Wisconsin-Madison, Madison, WI 53705 USA; 5 EBSCO Industries, Madison, WI 53726 USA; 6 School of Journalism and Mass Communication, University of Wisconsin-Madison, Madison, WI 53706 USA

**Keywords:** Breast cancer screening, Clinical decision support tool, Computational text analysis, Decisional satisfaction, Shared decision-making

## Abstract

**Background:**

Clinical decision aids may support shared decision-making for screening mammography. To inform shared decision-making between patients and their providers, this study examines how patterns of using an EHR-integrated decision aid and accompanying verbal patient-provider communication predict decision-making satisfaction.

**Methods:**

For 51 patient visits during which a mammography decision aid was used, linguistic characteristics of patient-provider verbal communication were extracted from transcribed audio recordings and system logs automatically captured uses of the decision aid. Surveys assessed patients’ post-visit decisional satisfaction and its subcomponents. Linear mixed effects models assessed how patients’ satisfaction with decision making was related to patterns of verbal communication and navigation of the decision aid.

**Results:**

The results indicate that providers’ use of quantitative language during the encounter was positively associated with patients’ overall satisfaction, feeling informed, and values clarity. Patients’ question-asking was negatively associated with overall satisfaction, values clarity, and certainty perception. Where system use data indicated the dyad had cycled through the decision-making process more than once (“looping” back through pages of the decision aid), patients reported improved satisfaction with shared decision making and all subcomponents. Overall satisfaction, perceived support, certainty, and perceived effectiveness of decision-making were lowest when a high number of navigating clicks occurred absent “looping.”

**Conclusions:**

Linguistic features of patient-provider communication and system use data of a decision aid predict patients’ satisfaction with shared decision making. Our findings have implications for the design of decision aid tools and clinician training to support more effective shared decision-making for screening mammography.

**Supplementary Information:**

The online version contains supplementary material available at 10.1186/s12911-022-02058-3.

## Background

Each year in the U.S., approximately 237,000 women are diagnosed with breast cancer, and about 41,000 women die from the disease [1]. Mammography has substantially improved breast cancer survival rates, but also presents risks, including false positives, anxiety, and over-diagnosis [2, 3]. Weighing benefits and risks of screening mammography is especially challenging among average risk women aged 40–49, who have lower breast cancer incidence and higher false positive rates [4]. Even when patients are motivated to make careful screening decisions, weighing numerous relevant factors can be daunting for patients and providers [5, 6]. The complexity of these decisions has prompted national guidelines that encourage shared decision making (SDM) [7], a bi-directional process in which both the patient and provider contribute to reaching a decision that reflects a patient’s risk profile as well as personal preferences [8]. However, guidelines do not specify mechanisms to operationalize the recommendation to perform SDM.

Though SDM may bring many benefits [9], it presents challenges in practice [10–12], requiring busy clinicians to elicit patients’ concerns and preferences regarding screening and deliver complex information that patients may struggle to comprehend, often involving numerical risks and technical terms [13–17]. A range of technology-supported clinical decision aids have emerged to facilitate SDM [18]. Such tools can help providers effectively convey information and help patients to participate actively in SDM [19]. These tools may present personalized risk and benefit information through interactive visualizations, help patients understand technical terms, and prompt clarification of patients’ personal values [20].

Prior work has categorized decision support aids into several types, including patient decision aids that can be used independently (i.e., ahead of or after clinical encounters), those that digitally mediate interactions between a provider and patient, and those used in face-to-face clinical encounters [21, 22]. While a number of studies have focused on independently-used patient decision aids [22], this paper focuses on tools used in face-to-face settings that are navigated by the provider, which we refer to as Clinical Decision Support Tools (CDSTs). Using CDSTs can focus both the patient and provider’s attention to the information being weighed in the decision process, and allow for concurrent verbal communication by providers and patients to reach a decision [21]. Thus far, evidence points to mixed effectiveness of using CDSTs in clinical encounters to support SDM. Some studies show that the use of CDSTs is associated with higher encounter ratings [23], increased patient knowledge [20, 24], decreased cancer-related distress [25], and reduced surgery costs [14]. In contrast, other studies find no link between use of CDSTs and patients’ knowledge [26], distress [27], or participation in SDM [28].

The inconsistent effectiveness of CDSTs may reflect, in part, how providers use these tools within encounters. While limited work has assessed how use of CDSTs affects the encounter, related work has examined use of electronic health records (EHR) during clinical encounters. These studies suggest that increased navigation of the EHR via mouse clicks can lead to provider fatigue and cause medical errors [29], and may result in less patient-centered communication and involvement as providers divide attention between the computer and patient [29, 30]. However, specific uses of CDSTs may underlie more effective SDM processes. For example, providers may backtrack in the tool (i.e., perform a ‘loop’) to run through different scenarios relating to the decision-making process. Such looping might capture the extent to which patients and providers engage purposefully with the tool and work through different possible risks and benefits. Since SDM is characterized by consideration of different options and outcomes [11], looping might allow providers and patients to examine a greater number of possible consequences, facilitating patients’ understanding of medical options, and, in the process, clarifying their own preferences.

Effective verbal communication between provider and patient is also a central component of clinical encounters. Several studies examine the linguistic features of provider communication, finding associations with patient satisfaction and outcomes [31–34]. However, linguistic features of verbal interactions have not been examined in the context of mammography SDM, or when using a CDST. Verbal communication may complement the interaction with the CDST, allowing the provider to respond to emergent patient concerns by elaborating, reiterating, or clarifying the information conveyed by the tool.

This study seeks to provide an initial understanding of how patterns of interaction, considering how both patient-provider verbal communication and navigation of the CDST are associated with women’s satisfaction with SDM. We examine these questions in the context of a CDST supporting mammography SDM that has been embedded in the EHR within a healthcare system and available to all providers as a resource to support mammography decision making since 2016. Our approach may inform future research examining heterogeneity in CDST use and its association with SDM outcomes. Findings may also suggest considerations related to the design of decision aids, and help identify areas where providers may need more training and support in enacting an effective technology-supported SDM process to help women make complex cancer screening decisions.

### Research questions and hypotheses

Our analyses are guided by a series of research questions and hypotheses about the ways SDM outcomes may relate to provider-patient verbal communication and CDST navigation.

As far as the effective elements of provider-patient verbal communication, prior work suggests that patient satisfaction is positively associated with both the volume of communication between provider and patient, and with the frequency of questions from patients [31, 35–37]. In the context of SDM, patient word count may suggest active involvement in the discussion, which may translate to feeling more satisfied that decisions reflect their preferences and values [27, 32]. Likewise, asking more questions may reveal participation in SDM, highlighting areas where the patient lacks understanding and eliciting additional explanation to satisfy informational needs [38, 39]. Providers’ “affect words” reference positive or negative moods or mindsets (e.g., nice, sweet, sad, worried). In the context of mammography decision-making, such words may demonstrate providers’ attentiveness to the personal effects of medical decision-making, with such consideration potentially influencing rapport and partnership during medical visits and increasing SDM satisfaction. Moreover, quantifier words (e.g., few, many, much) are essential for clear presentation of potential consequences associated with different care plans, and a critical part of informed decision making [13].

Next, we examine the impact of CDST navigation, measured by clicks (selecting or navigating between elements of the tool’s interface) and loops (backtracking in the tool to revisit a prior page). The implications of clicks are unclear in the context of a CDST for SDM, where clicks may also serve to uncover personalized information but might also be associated with fatigue, errors, and distraction. Loops may reflect a more directed type of use, allowing providers to work with patients to understand the implications of varying decisions and their consequences, helping patients to clarify their values and preferences, and ultimately supporting SDM satisfaction. Finally, the relationship between clicks and loops is unclear. Thus, we examine whether the potential association between clicks and SDM satisfaction may change as a function of whether those clicks are devoted to looping, or to other activities within the tool.

We propose the following hypotheses and research questions:

H1a & b: Overall word count (a) and more patient questions (b) will be positively associated with SDM satisfaction.

- RQ1 & 2: What is the relationship of providers’ use of affect words [1] and quantifier words [2] with SDM satisfaction?
- RQ3: What is the relationship between clicks in a CDST and SDM satisfaction?
- RQ4: What is the relationship between looping in a CDST and SDM satisfaction?
- RQ5: Is there an interaction between clicks and loops on SDM satisfaction? (i.e., Does the relationship between clicks and SDM satisfaction vary based on the number of loops?)

## Methods

### Study context

This study examined these issues in the context of a CDST called the Breast Cancer Risk Estimator-Decision Aid (BCaRE-DA), a software platform designed for collaborative use by patients and providers to support informed breast cancer screening decisions, and emphasizing average risk women aged 40–49, for whom these decisions can be particularly challenging [23, 40]. BCaRE-DA seeks to help the patient-provider dyad engage in a thorough, systematic, and interactive process in which they touch on factors including women’s family history, race, ethnicity, and breast density, and enter these data into the tool to allow personalization of an individual’s risk profile. In creating the CDST, a multi-disciplinary design team relied on input from women aged 40–49, and from providers experienced in supporting mammographic decision-making.

While verbally communicating with a patient, a provider clicks through a series of pages in the BCaRE-DA, first entering individual data and the patient family history, then viewing personalized risk information, and ultimately making a collaborative decision. BCaRE-DA prompts a chronological sequence of activities. The provider moves sequentially through several pages: data entry, baseline assessment, and screening decision options. First, on the “Data” page (Additional file 1: Fig. 1a), age and other information gathered from the electronic record are pulled into the tool and can be reviewed and corrected with the patient’s input. On the “Assessment” page, the patient’s estimated risk of breast cancer development in the next 10 years is displayed numerically or as a bar graph, as well as guideline recommendations (Additional file 1: Fig. 1b). On the “Decision” page, the provider and patient can see a visualization of the likelihood of possible outcomes if they make a particular decision, i.e., to get a screening mammogram or not (Additional file 1: Fig. 1c). Patients and providers may also return to prior pages to edit data, enter additional data, and to refresh or review personalized risk and benefit information.

**Fig. 1:**
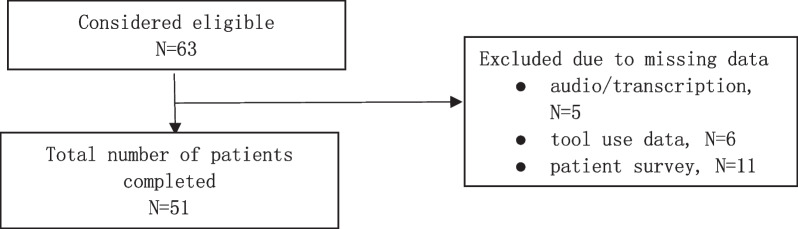
Diagram of patient sample collection (Some participants have more than one missing data point)

The BCaRE-DA tool was first implemented in the EHR in May 2016, with its uptake supported by distributing a reference document to primary care providers that illustrated how to access the tool within the EHR, and through providers discussing the tool and recommending it to colleagues. The tool developers tracked tool use over time. Utilization of the tool increased slowly during the time prior to this study, which was conducted between May 2017 and May 2018. A limited number of primary care providers who were already using the tool (early adopters) and who had a patient panel meeting the inclusion criteria (women 40–49) were invited to participate. Eleven providers agreed to participate and provided informed consent. Their female patients aged 40–49 who had an average risk of developing breast cancer were invited to the study if they scheduled a primary care appointment with these providers in which a discussion of mammography screening would be appropriate. During the visit, providers used the tool to guide a discussion leading to a decision regarding screening mammography. These discussions were audiotaped and transcribed, and all page views within the BCaRE-DA tool were automatically recorded in a system use log. Within a week after the visit, patients were mailed a survey to gauge satisfaction with various elements of SDM. The study was approved by the institution’s Institutional Review Board.

### Participants

Eligible participants had no history of dementia or breast cancer, spoke English as their primary language, and were willing to use the BCaRE-DA tool to support their decision-making regarding screening mammography and to have their interactions audio recorded and logged by the BCaRE-DA tool. While women were eligible whether or not they had past mammography experience, those who had a mammogram in the nine months preceding their scheduled visit were excluded as they would not yet be eligible for their next mammographic screening. Sixty-three eligible patient participants provided informed consent. We had complete data for 51 of these patients (analyzed here), comprising the audio transcription, completed post-visit patient survey, and system use logs for the BCaRE-DA tool. Figure 1 summarizes data completeness for the 63 eligible participants. All records were merged based on patients’ and clinicians’ unique identifiers, which were recorded in all data files.

### Linguistic dimensions of verbal communication

Audio recordings of the study visits were transcribed by a professional transcriptionist. Transcripts were then automatically coded for linguistic dimensions of providers’ and patients’ verbal contributions during the encounter using the Linguistic Inquiry and Word Count (LIWC) software [41], a dictionary-based text analysis program. LIWC calculates the percentage of words in a segment of text that fall into pre-defined linguistic categories (i.e., the count of words that fall in a linguistic category, divided by the count of total words). For example, a provider who used 20 affect words out of 500 words uttered during the visit would have an affect word score of 4%. Following from our research questions and hypotheses, the analyses included five linguistic categories: total number of words used by each speaker (patient or provider), patient questions, provider affect words (e.g., happy, worried), and provider quantifiers (e.g., few, many) (for more examples, see Pennebaker et al., 2007).

### Survey data

*Overall Decisional Satisfaction* was measured using the *Decisional Conflict Scale (DCS)* developed by O’Connor [42].The measure includes 15 items that were rated on a 5-point scale ranging from 0 (*Strongly Agree*) to 4 (*Strongly Disagree*). The formula defined by O’Connor calls for items to be summed, divided by 15, and multiplied by 25, such that total scores range from 0 to 100. For ease of interpretation, decisional conflict scores were reverse coded such that higher scores indicate higher satisfaction with SDM, whereas lower scores represent lower satisfaction with SDM [43].

Our study also examined five subscales of the DCS,^1^ representing patients’ perceptions that: 1) they were well informed when making their decision (“informed decision”); 2) they understood which risks and benefits matter most to them (“values clarity”); 3) they received adequate support and/or advice (“support perception”); 4) they felt confident that their choice was best for them (“certainty perception”); and 5) they felt their decision was effective (“effective decision”). Subscale scores were summed, divided by the number of items, multiplied by 25 [42], and reverse coded to ease interpretation.

*Covariates/control variables* were years of education and prior mammography experience. The level of education may affect SDM by influencing patients’ confidence to participate in SDM or ability to process complex or technical information. We recoded patients’ highest completed education level into a continuous variable by estimating the total years of education corresponding to each categorical response (e.g., those with some college or a 2-year degree were assigned a value of 14 years, and those with a 4-year degree were assigned a value of 16 years, etc.). Prior mammography was a binary variable capturing whether patients had ever had a mammogram. As participants’ eligibility for the study was based on their age and being at average risk of breast cancer, we did not control for age or for aspects of patients’ medical history.

### System use data

The CDST automatically recorded system navigation in usage logs. Two variables were extracted from the logs: 1) the number of mouse clicks, and 2) the number of loops. We define a loop as returning to any previous page in the sequence. For example, one patient’s record might show two loops, one where the clinician navigated from the Assessment page back to the Data page, and then later from the Decision page back to the Assessment page. Such loops allow clinicians to re-enter or edit patient data and to re-populate personalized recommendations or adjust how they are visualized, thus allowing for testing alternative scenarios, further processing information, or simply confirming and reinforcing the decision.

### Data analysis

Providers may each have a unique approach to facilitating SDM. To take account of the effects of providers’ differing styles, we conducted a series of linear mixed-effect models wherein patients were nested within clinicians. The use of linear mixed-effect models can provide standard errors corrected for non-independence in the data and information about effects within and between groups [44]. We controlled patients’ years of education and prior mammography experience as covariates when examining the relationship between linguistic and system use features and study outcomes.

We ran separate models predicting overall decisional satisfaction and each of its five subscales in relation to language use and system use data. The independent variables in the fixed effects were: provider word count, patient word count, patient question asking, provider affect words, provider quantifier words, clicks, loops, and the click by loop interaction (only entered in model 2). Covariates were patients’ years of education and prior mammography. Provider identifier was entered as a random effect.

## Results

### Patient profile

The mean age of participants was 44.1 years (SD = 2.7). As far as the highest completed level of education, 7.8% had some high school or a high school diploma, 20.4% had some college or 2-year degree, 38.9% had a 4-year college degree, and 33.3% had completed more than a 4-year college degree. Forty-five of the 51 participants (88.2%) reported having mammography before. All had health insurance.

### Provider profile

Ten female clinicians and one male clinician working at the study University participated in this study. Their ages ranged from 37 to 63 years old, and they had 9–36 years in practice in primary care. Three were physicians from Internal Medicine, six were physicians from Family Medicine, and two were nurse practitioners from Ob/Gyn. Providers conducted between 2 and 9 visits each as part of this study. Table 1 presents the descriptive linguistic features, system log data, and measures from the survey.

**Table 1:** Descriptive table for linguistic features, system use and survey measures

	Mean	SD	Cronbach’s α
Provider word count	916.4	381	
Patient word count	192.8	160.23	
Patient question marks	1.24	1.26	
Provider affect words	4.07	0.99	
Provider quantifier	3.95	0.75	
Number of mouse clicks	11.61	7.09	
Number of loops	0.51	0.73	
Overall decisional satisfaction	44.92	11.79	0.93
Feeling informed	40.03	14.24	0.82
Values clarity	51.72	18.03	0.82
Support perception	45.92	9.77	0.69
Certainty perception	62.75	18.28	0.82
Effective decision	37.38	11.29	0.84

### Main effects

H1a predicted that word counts contributed verbally by providers and patients would be associated with SDM satisfaction, but this hypothesis was not supported, as patient and provider word use were not significantly associated with study outcomes. H1b predicted that question-asking from patients would be associated with SDM satisfaction, but this was also not supported and, contrary to our expectations, asking more questions was negatively associated with patients’ overall satisfaction (β =  − 0.32, *p* = 0.01) (Table 2), values clarity (β =  − 0.39, *p* = 0.001) (Additional file 1: Table 4), and certainty perception (β =  − 0.32, *p* = 0.001) (Additional file 1: Table 6).

**Table 2:** Linear mixed-effect model of linguistic features and system use predicting patients’ overall decisional satisfaction

	Model 1 (only main effects)			Model 2 (interaction effects)		
	β	SE	*p* value	β	SE	*p* value
*Patient demographics*						
Patient’s years of education	− 0.21	0.12	0.08	− 0.17	0.12	0.12
Mammography history (No = 0; Yes = 1)	− 0.12	0.36	0.74	0.09	0.33	0.80
*Linguistic features*						
Provider word count	0.14	0.13	0.28	0.08	0.12	0.49
Patient word count	− 0.11	0.13	0.38	− 0.12	0.12	0.34
Patient question marks	− 0.32**	0.12	0.01	− 0.40***	0.12	0.001
Provider affect words	− 0.11	0.13	0.40	− 0.06	0.12	0.60
Provider quantifier words	0.30**	0.12	0.01	0.23*	0.11	0.04
*System use*						
Total clicks	− 0.27*	0.12	0.02	− 0.31**	0.11	0.000
Loops	0.34*	0.12	0.004	0.37	0.11	0.22
*Interaction*						
Loops* Total clicks				0.40**	0.13	0.003
*Constant*	0.10	0.33	0.000	− 0.13	0.31	0.000
Random effects						
Between-provider variance	− 24.63	7.99		− 24.25***	5.91	
Log likelihood	− 56.91			− 52.73		
Wald Chi2	40.67			56.98		

*p < .05. **p < .01. ***p < .001

RQ1 examined the relationship between provider affect words and SDM satisfaction. Provider affect words were not associated with study outcomes.

RQ2 investigated the relationship between provider quantifier words and SDM satisfaction. Provider use of quantifier language was significantly positively associated with overall satisfaction (β = 0.30, *p* = 0.01) (Table 2), feeling informed (β = 0.37, *p* = 0.002) (Additional file 1: Table 3), and values clarity (β = 0.33, *p* = 0.01) (Additional file 1: Table 4).

RQ3 investigated the relationship between clicks and SDM satisfaction. Total clicks were negatively associated with overall satisfaction (β =  − 0.27, *p* = 0.02) (Table 2), feeling informed (β =  − 0.25, *p* = 0.04) (Additional file 1: Table 3), support perception (β =  − 0.30, *p* = 0.03) (Additional file 1: Table 5), and certainty perception (β =  − 0.34, *p* = 0.01) (Additional file 1: Table 6).

Finally, RQ4 investigated the relationship between looping and SDM satisfaction. Our results indicated that looping back through CDST pages was positively associated with overall decisional satisfaction (β = 0.34, *p* = 0.004) (Table 2), feeling informed (β = 0.24, *p* = 0.04) (Additional file 1: Table 3), values clarity (β = 0.26, *p* = 0.03) (Additional file 1: Table 4), support perception (β = 0.36, *p* = 0.01) (Additional file 1: Table 5), certainty perception (β = 0.26, *p* = 0.03) (Additional file 1: Table 6), and effective decision-making (β = 0.34, *p* = 0.01) (Additional file 1: Table 7).

### Interaction effects

**Fig. 2:**
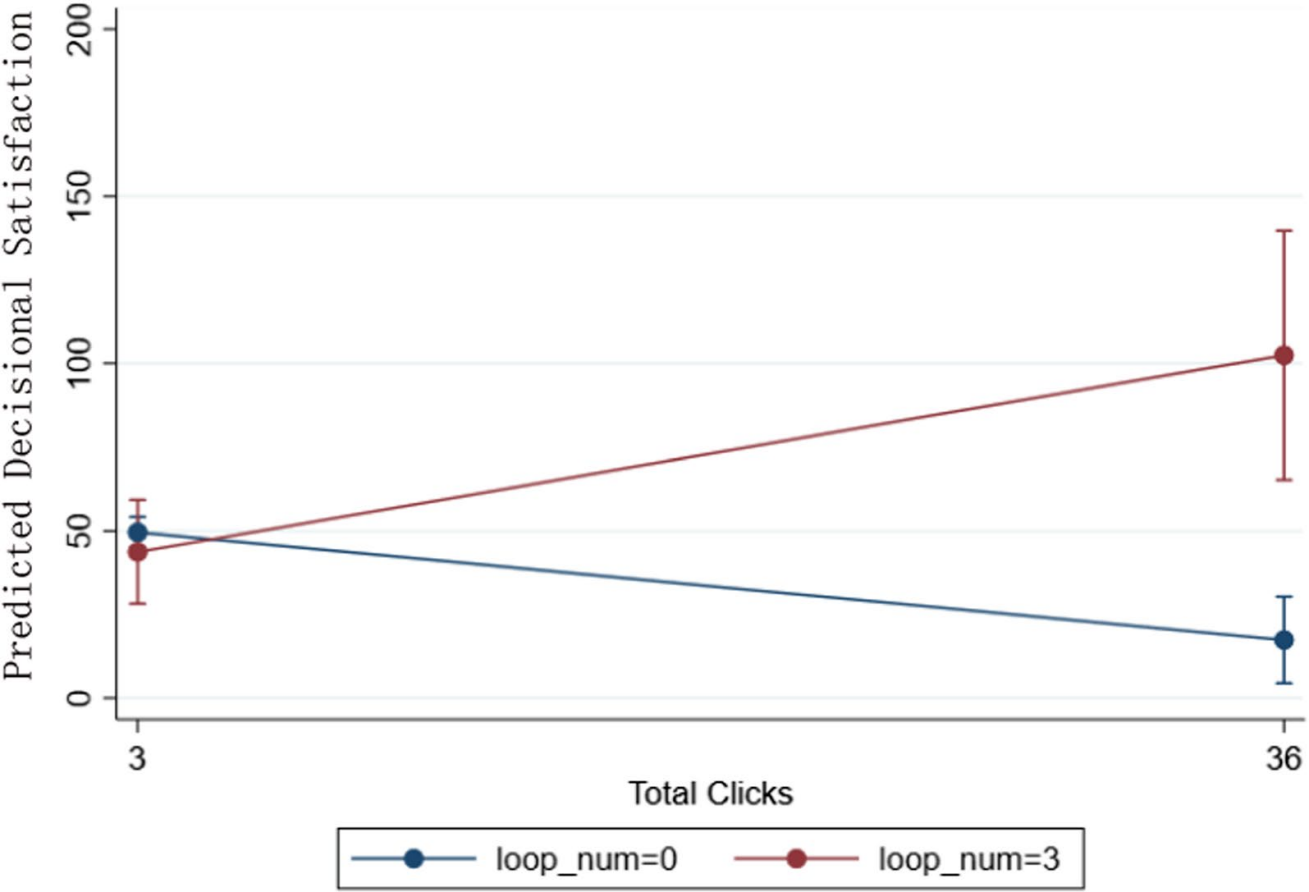
Interaction effect of clicks and loops on overall decisional satisfaction. *Note* For illustration purpose, this plot represents the predicted value of the overall decisional satisfaction

We finally examined the interaction effects between clicks and looping on SDM satisfaction (RQ5). We found significant interactions between clicks and looping such that, when click count was higher in the presence of more loops, patients felt more satisfied overall (Loops*clicks: β = 0.40, *p* = 0.003, see Table 2; Fig. 2), more supported (Loops*clicks: β = 0.43, *p* = 0.0001, see Additional file 1: Table 5; Additional file 1: Fig. 2a), more certain (Loops*clicks: β = 0.42, *p* = 0.003, see Additional file 1: Table 6; Additional file 1: Fig. 2b), and perceived greater effectiveness of the decision (Loops*clicks: β = 0.50, *p* = 0.001; see Additional file 1: Table 7 and Additional file 1: Fig. 2c); these ratings were especially poor when click count was higher in the absence of looping.

## Discussion

This study examined how satisfaction with decision-making around screening mammography was related to navigation patterns of a CDST and concurrent linguistic features of patient-provider verbal communication. Findings from our study suggest that linguistic dimensions of the encounter and behavioral data gathered in CDST log files are both associated with patients’ SDM satisfaction. Specifically, whereas the amount of communication (i.e., speaker word count) and provider affect words showed no association with satisfaction, patient satisfaction was associated with the rate at which providers used quantitative language. Patient question asking was negatively associated with overall decision-making satisfaction. Regarding CDST use, our findings suggest that more clicks within the tool were negatively associated with SDM satisfaction, whereas looping back through pages in the tool was positively associated with SDM satisfaction. Moreover, patients felt more satisfied when high click counts occurred with looping, whereas patients felt less satisfied when high click counts occurred without looping.

### Linguistic features

These findings are consistent with prior studies in highlighting the importance of quantitative information in facilitating decision-making [15, 16]. Many CDSTs, including the tool evaluated in this study, describe breast cancer risks with precise numbers or graphical presentations, which can help patients understand probabilities [9, 14] and make informed decisions [15]. Our study suggests that providers’ verbal communication may also play an important complementary role in SDM, with the use of quantifiers likely reflecting efforts to reinforce the quantitative risk information relayed in the CDST, helping patients better understand screening options. While our study provides preliminary evidence of the importance of using quantifiers in SDM accompanying CDST use, future study may explore the effectiveness of specific types of quantitative information, as prior work suggests that different ways of communicating numbers (e.g., odds ratio, absolute risk difference, relative risk) are associated with differing levels of comprehension and satisfaction [33, 34].

Other findings are not consistent with the prior literature. Past studies suggest positive associations between word count, as a proxy of depth of the interaction, and patient satisfaction, whereas we did not find any such association. However, past studies have not been conducted in the context of using CDSTs, and it is possible that word count might work differently in this context. For example, gestures may play a role in communication when using a CDST (e.g., pointing to information or buttons on the screen), relatively brief utterances could prompt important actions in the tool (e.g., requests to run through the sequence again), or too much verbal communication concurrent with tool use could disrupt or distract processing information conveyed in the tool. This interplay of communication volume and tool use warrants further research.

Likewise, regarding affect words, we did not find a significant relationship to SDM satisfaction, which could perhaps reflect the sample in this study, with most patients having completed mammography before. Most patients therefore have prior experience weighing potential affective consequences of outcomes like false positives or cancer diagnoses. For such patients, perhaps discussing up-to-date quantitative information that clarifies their personal risks and benefits from mammography is of greater value.

Surprisingly, whereas we had hypothesized that question asking would indicate patients’ active involvement in SDM and associate with higher satisfaction, we found that patients who asked more questions tended to feel less satisfied overall, less clear about their values, and less certain about their decision at follow-up. These negative associations need to be further explored but could suggest that information was not clearly conveyed, or that clinicians did not adequately address patients’ questions. Prior studies showed that the extent to which patients ask questions is contingent on the clinicians’ communication style [45]. Moreover, patients who receive adequate answers from their clinicians show better psychological adjustment than those who do not [46]. A review of transcripts revealed that patients asked a variety of questions seeking to clarify information presented by the tool (e.g., "What is normal? What are the other normal timelines?"), as well as questions related to issues that were not addressed in the tool, such as insurance coverage (e.g., “I don't think insurance pays for them yearly—they do?"), and coordination and scheduling of mammography (e.g., “Is there any way to coordinate getting that with having to do a physical or some other reason that I'm coming in… So—and I can coordinate those two visits?”). However, further study is needed to systematically examine the types of questions patients ask and the adequacy of answers they receive.

### Tool use

Our findings also suggest that looping in a CDST may play an important role in SDM. Specifically, we found that the relationship between clicks within the CDST and SDM satisfaction varied based upon looping in the system. With fewer clicks, SDM outcomes were not related to the extent of provider looping in the CDST. However, with more clicks, patients reported better SDM outcomes when clinicians looped through more scenarios and worse outcomes without looping. This finding may relate to prior work showing that more clicks in the EHR are associated with lower patient satisfaction [29, 30]. Our results show that clicks may not be helpful even if they ostensibly occur in the context of providers and patients navigating a CDST together, perhaps because the provider lacks knowledge of how to efficiently use the tool, or because certain types of engagement with navigating the tool may distract from patient-centered communication. However, directed use of the CDST, such as when clicks occurred as providers looped through multiple scenarios, may help patients feel more satisfied, more supported, and perceive more effectiveness about their decision.

To improve the process of navigating CDSTs, it may be important to identify where and when clicks occur in the tool. Higher clicks may be important markers of a challenging decision process or a provider’s lack of experience with the tool [47], perhaps capturing struggles to make sense of the information provided or find the best choice.

### Practical implications

These findings can inform SDM provider training to optimize CDST use in clinical encounters. There may be value in providing proper training before large scale implementation of SDM supported by CDSTs, as it is possible that inexperience with a CDST could lead to inefficient use that detracts from SDM. Moreover, it may be beneficial to educate clinicians on verbal communication skills relevant to SDM, such as how to verbalize information in quantitative terms (e.g., risk and associated outcomes) to accompany the visual displays in the CDST, provide adequate answers to questions, and verbally engage patients while simultaneously managing data in the CDST [48]. Moreover, clinicians’ training could offer additional experience and guidance with interface elements to reduce unnecessary clicks and save valuable time during the visit.

These findings also have implications for the design of CDSTs. To improve patient satisfaction, the digital interface must be user-friendly for the patient as well as the clinician [47, 48]. Our study suggests looking to click counts to potentially identify specific user interface elements where the CDST could be improved to further ease navigation. Designers may also consider how the tool can help patients to raise questions and find satisfactory answers. These findings also suggest potential opportunities to automatically monitor encounters and to intervene on possible decisional conflict. For example, if a provider’s use of the CDST involves many clicks but few loops, the CDST creator could provide additional resources or tips for navigating the tool.

### Limitations and directions for future research

Our study has several limitations. First, we cannot draw conclusions about the causal relationships among the measured variables. We assessed relationships between linguistic patterns and tool use and SDM satisfaction while controlling for potential confounding variables, but there may be additional unmeasured factors that contribute to the observed relationships. For example, patients’ health literacy should likely be a covariate [49].

Second, more work may be needed to distinguish activities in the CDST that predict SDM in different ways, including examining when and why clinicians clicked on different parts of the interface, and whether certain patterns drove the negative association between click count and patients’ SDM satisfaction. There may also be particular types of loops that are helpful or unhelpful, based on the specific scenarios explored.

Additional limitations relate to generalizability. As the participants are all from one geographic region, it would be beneficial to replicate this study in other locations. Our study also has a large proportion of patients who had a college education or higher, which may limit the ability to generalize to others. Our patient sample was also entirely women, and all but one of the providers in our study were women. Past work suggests that gender can shape clinical interactions, and it would likely also have implications for how decision aid tools can be effectively used. For example, some work suggests that women are more motivated than men to participate actively in SDM [50], and that they may have higher information needs during SDM than men [50]. In addition, patterns of verbal communication during clinical encounters are known to vary based on the gender dynamics of the provider-patient dyad, with some research suggesting that female/female dyads are, on average, more verbally communicative during visits [51]. These patterns of findings may be consistent with a high importance of concurrent verbal communication to reinforce CDST use among women, including disambiguating and elaborating on the information a tool provides. Furthermore, since the providers in this study were early adopters, future work may examine uses of CDSTs and SDM outcomes when providers have less experience with CDSTs or are slower to adopt them. Finally, our study examined tool use in the absence of formal training, and future studies may examine whether formal training (which has been developed for BCaRE-DA since data collection) could potentially change use patterns and SDM satisfaction.

As far as future directions, time is an important element to consider in relation to any CDST. Time in clinical settings can be understood as the available length of consultation time and the time constraints perceived by the provider and the patients [32]. Limited time can result in providers being more directive and less likely to encourage patients to ask questions [32], and some scholars and practitioners argue that time constraint is therefore the main barrier to SDM [32]. Thus, CDSTs that can efficiently support SDM are much needed. The present research did not consider the association between time using the tool and SDM satisfaction due to data limitations, nor did it consider its connection to total word count, but this is a high priority area for future investigation. If the CDSTs are helpful in SDM but take too much time to use in the context of clinic visits, one solution may involve promoting tool use outside of clinical visits, when patients have extra time to navigate and reflect on their decisions. For instance, future work might consider complementary tools or modules that a patient can use independently. Before a clinical encounter, such tools could summarize key information and allow for patients to identify questions and prioritize discussion topics to cover in clinical encounters [21]. After an encounter, such tools could provide methods to seek further information or to connect with peers or professionals for social support [21, 52].

Very limited work has addressed heterogeneity in uses of CDSTs, and how this predicts satisfaction with decision-making. As such, this study provides an important but preliminary step toward understanding these issues. Future work should seek to confirm these findings with a larger sample size that provides increased power.

## Conclusions

As CDSTs are increasingly deployed to support SDM, it is critical to understand how specific uses of these tools, and the accompanying patient-provider communication, affect SDM satisfaction. This study suggests that elements of both system navigation and verbal communication are associated with women’s post-visit satisfaction with SDM, suggesting areas for future study, as well as implications for the design of CDSTs and for training clinicians in effective use of these tools.

### Supplementary Information

**Additional file 1**. **Figure S1a.** ‘Data’ page from the Breast Cancer Risk Estimator Decision Aid. **Figure S1b**. ‘Assessment’ page from the Breast Cancer Risk Estimator Decision Aid.**Figure S1c**. ‘Decision’ page from the Breast Cancer Risk Estimator Decision Ai. **Table S3**. Linear Mixed-effect Model of Linguistic Features and System Use on Patient’s Feeling Informed. **Table S4**. Linear Mixed-effect Model of Linguistic Features and System Use on Patient’s Benefit/Risk Clarity. **Table S5**. Linear Mixed-effect Model of Linguistic Features and System Use on Patient’s Support/Advice Perception. **Table S6**. Linear Mixed-effect Model of Linguistic Features and System Use on Patient’s Value Clarity. **Table S7**. Linear Mixed-effect Model of Linguistic Features and System Use on Patient’s Confidence/Certainty about Decision Making. **Figure S2a**. Interaction effect of clicks and loops on patient support/advice perception. **Figure S2b**. Interaction effect of clicks and loops on patient value clarity. **Figure S2c**. Interaction effect of clicks and loops on patient confidence/certainty about decision making.

## Data Availability

The datasets generated and/or analyzed during the current study are not publicly available due to human data, but are available from the corresponding author on reasonable request.

## Appendix: Items for subscales. All items were from Decisional Conflict Scale (DCS) developed by O’Connor [42]

**Table:**

**Feeling informed**
Do you know which options are available to you?
Do you know the benefit of each option?
Do you know the risks and side effects of each option?
**Clarity about risk and benefit**
Are you clear about which benefits matter most to you?
Are you clear about which is more important to you (the benefits or the risks and side effects)?
**Receiving adequate support and/or advice**
Do you have enough support from others to make a choice?
Are you choosing without pressure from others?
Do you have enough advice from others to make a choice?
**Clarity about value and preference**
Are you clear about the best choice for you?”
Do you feel sure about what to choose?
Is this decision easy for you to make?
**Confidence or certainty about their decision**
Are you satisfied with your decision?
Do you feel you have made an informed choice?
Does your decision show what is important to you?
Do you expect to stick with your decision ?

## Abbreviations

SDM: Shared decision making
CDST: Clinical decision support tools
LIWC: Linguistic inquiry and word count
BCaRE-DA: Breast cancer risk estimator-decision aid

## Footnotes

1 The questionnaire for the five dimensions of SDM satisfaction is presented in the “Appendix”.
