# Supplementary material for: Patient-provider communication while using a clinical decision support tool: explaining satisfaction with shared decision making for mammography screening
Source: BMC Med Inform Decis Mak. 2022 Dec 7;22:323. doi: 10.1186/s12911-022-02058-3 (PMC9730626; doi:10.1186/s12911-022-02058-3)
Supplement: Supplementary file 1 — Additional file 1. Figure S1a. ‘Data’ page from the Breast Cancer Risk Estimator Decision Aid. Figure S1b. ‘Assessment’ page from the Breast Cancer Risk Estimator Decision Aid.Figure S1c. ‘Decision’ page from the Breast Cancer Risk Estimator Decision Ai. Table S3. Linear Mixed-effect Model of Linguistic Features and System Use on Patient’s Feeling Informed. Table S4. Linear Mixed-effect Model of Linguistic Features and System Use on Patient’s Benefit/Risk Clarity. Table S5. Linear Mixed-effect Model of Linguistic Features and System Use on Patient’s Support/Advice Perception. Table S6. Linear Mixed-effect Model of Linguistic Features and System Use on Patient’s Value Clarity. Table S7. Linear Mixed-effect Model of Linguistic Features and System Use on Patient’s Confidence/Certainty about Decision Making. Figure S2a. Interaction effect of clicks and loops on patient support/advice perception. Figure S2b. Interaction effect of clicks and loops on patient value clarity. Figure S2c. Interaction effect of clicks and loops on patient confidence/certainty about decision making. [file 12911_2022_2058_MOESM1_ESM.docx]

Additional file 1

Figure S1a. ‘Data’ page from the Breast Cancer Risk Estimator Decision Aid


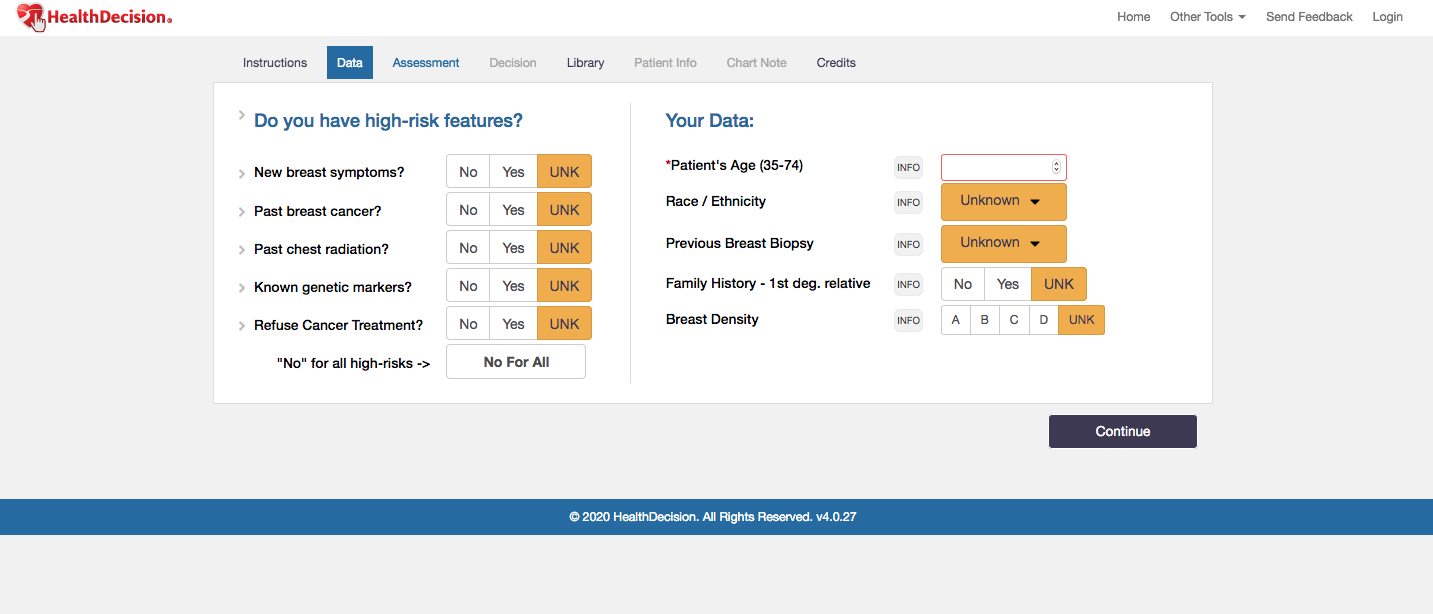


Figure S1b. ‘Assessment’ page from the Breast Cancer Risk Estimator Decision Aid


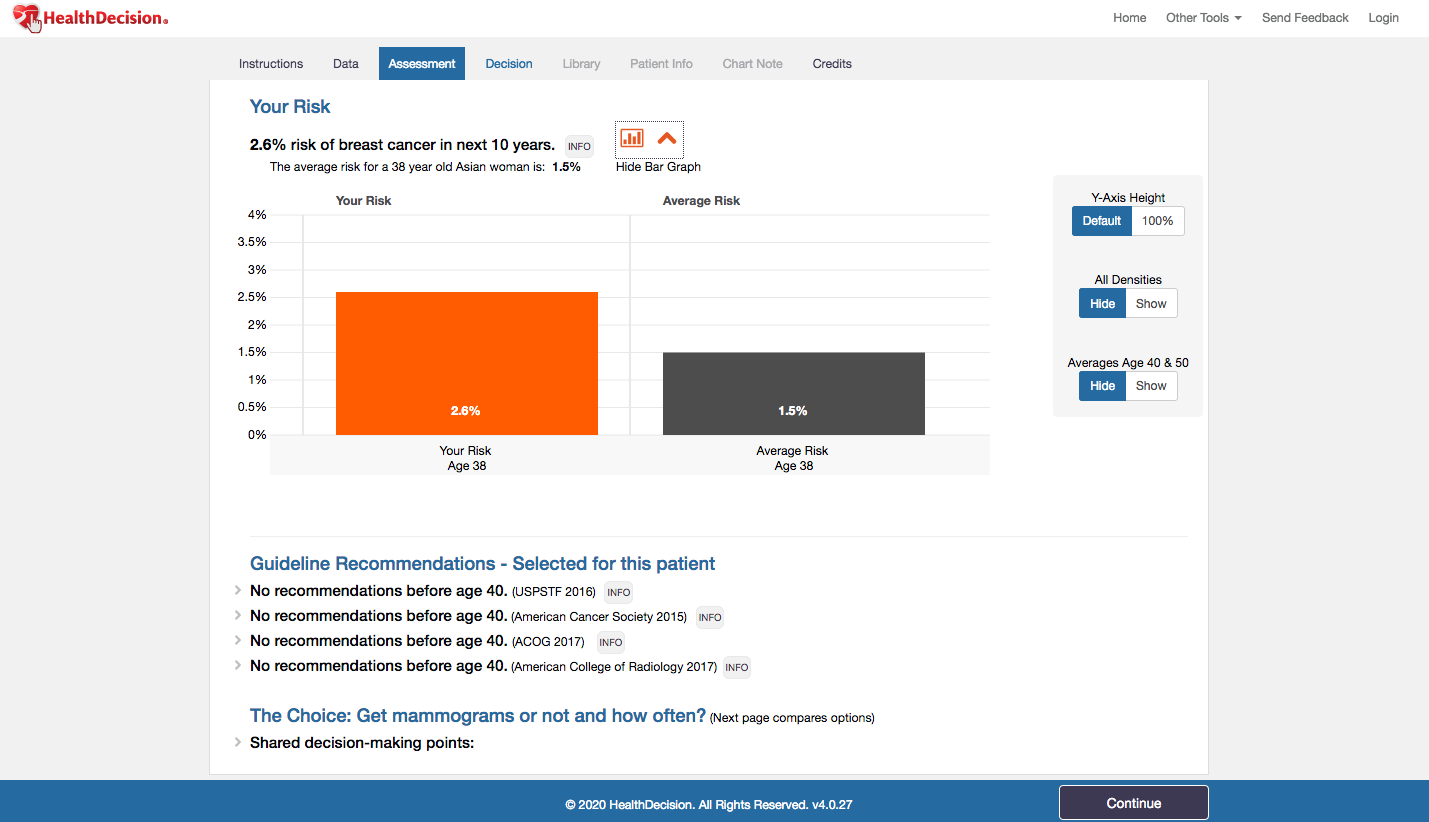


Figure S1c. ‘Decision’ page from the Breast Cancer Risk Estimator Decision Aid


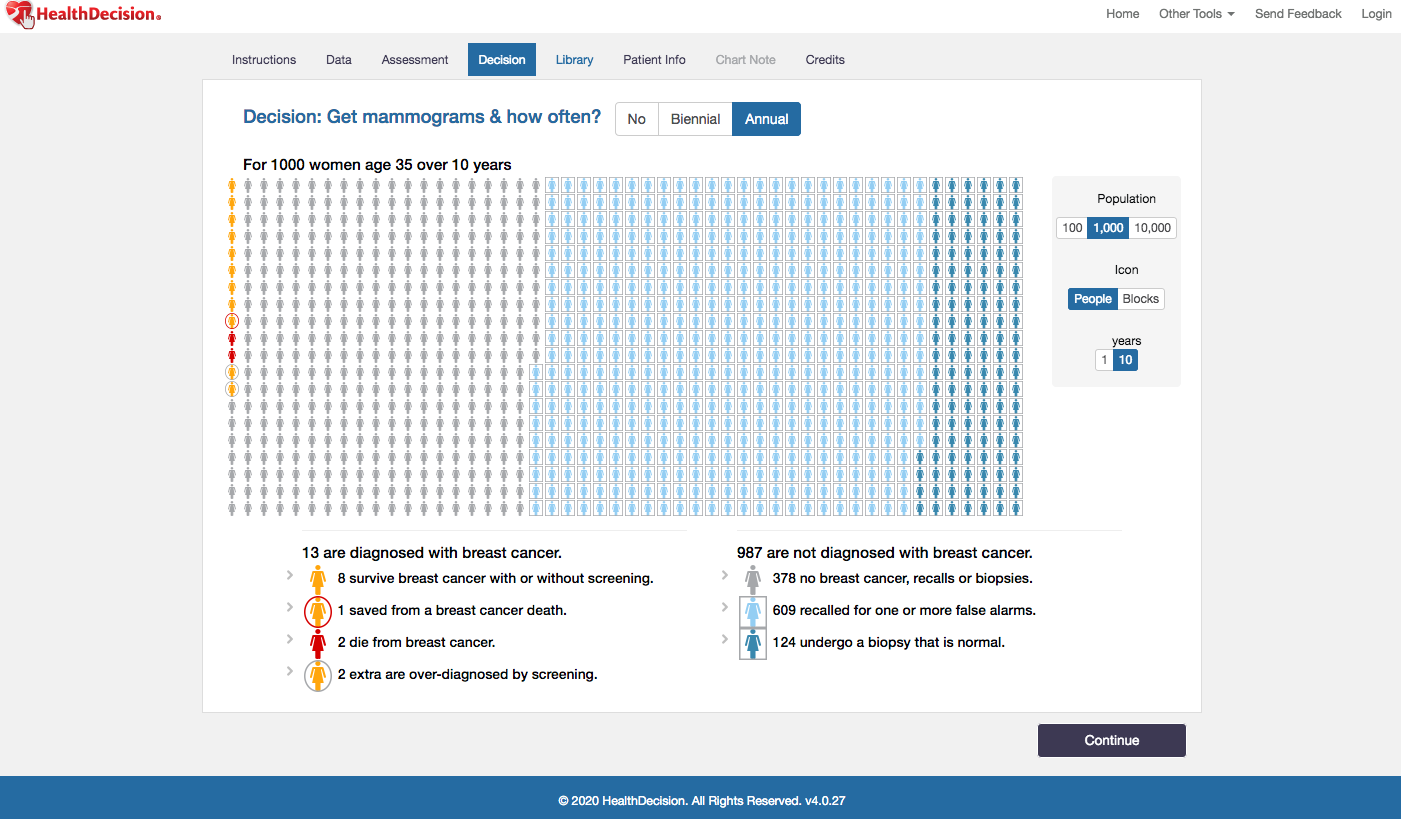


Table S3. Linear Mixed-effect Model of Linguistic Features and System Use on Patient’s Feeling Informed

|  | Model 1 (only main effects) | | Model 2 (interaction effects) | |
| --- | --- | --- | --- | --- |
|  | β | S.E | β | S.E |
| *Patient Demographic* |  |  |  |  |
| Age | -0.083 | 0.127 | -0.074 | 0.125 |
| Education | -0.260* | 0.121 | -0.223 | 0.122 |
| *Linguistic Features* |  |  |  |  |
| Provider word count | 0.201 | 0.141 | 0.18 | 0.14 |
| Patient word count | -0.026 | 0.143 | -0.03 | 0.14 |
| Patient question marks | -0.21 | 0.128 | -0.249 | 0.13 |
| Provider affect words | -0.059 | 0.132 | 0.03 | 0.132 |
| Provide quantifier | 0.349** | 0.124 | 0.318* | 0.124 |
| *System Use* |  |  |  |  |
| Total clicks | -0.265* | 0.121 | -0.279* | 0.119 |
| Loops | 0.273* | 0.12 | 0.282* | 0.118 |
| *Interaction* |  |  |  |  |
| Loops* Total clicks |  |  | 0.191 | 0.148 |
| Constant | 9.05E-09 | 0.107 | -0.025 | 0.107 |
| **Random effects** |  |  |  |  |
| Between-provider variance | 22.01*** | 6.006 | -16.75* | 7.37 |
| **Log Likelihood** | -57.11 |  | -56.3 |  |
| **Wald Chi2** | 35.22 |  | 38.03 |  |
| *Note: * p < .05. **p < .01. ***p < .001* | |  |  |  |

Table S4. Linear Mixed-effect Model of Linguistic Features and System Use on Patient’s Benefit/Risk Clarity

|  | Model 1 (only main effects) | | Model 2 (interaction effects) | |
| --- | --- | --- | --- | --- |
|  | β | S.E | β | S.E |
| *Patient Demographic* |  |  |  |  |
| Age | -0.157 | 0.124 | -0.152 | 0.124 |
| Education | -0.257* | 0.118 | -0.238* | 0.121 |
| *Linguistic Features* |  |  |  |  |
| Provider’s word count | 0.123 | 0.138 | 0.112 | 0.138 |
| Patient’s word count | -0.007 | 0.139 | -0.009 | 0.139 |
| Patient’s question marks | -0.341** | 0.125 | -0.361** | 0.128 |
| Provider’s affect words | -0.068 | 0.129 | -0.053 | 0.131 |
| Provider’s quantifier | 0.302* | 0.121 | 0.286* | 0.123 |
| *System Use* |  |  |  |  |
| Total clicks | 0.099 | 0.118 | -0.106 | 0.118 |
| Loops | 0.289* | 0.117 | 0.294* | 0.117 |
| *Interaction* |  |  |  |  |
| Loops* Total clicks |  |  | 0.097 | 0.147 |
| Constant | 1.89E-08 | 0.105 | -0.013 | 0.106 |
| **Random effects** |  |  |  |  |
| Between-provider variance | -23.92** | 7.526 | 23.70*** | 6.621 |
| **Log Likelihood** | -55.96 |  | -55.74 |  |
| **Wald Chi2** | 39.24 |  | 40.02 |  |
| *Note: * p < .05. **p < .01. ***p < .001* | |  |  |  |

Table S5. Linear Mixed-effect Model of Linguistic Features and System Use on Patient’s Support/Advice Perception

|  | Model 1 (only main effects) | | Model 2 (interaction effects) | |
| --- | --- | --- | --- | --- |
|  | β | S.E | β | S.E |
| *Patient Demographic* |  |  |  |  |
| Age | -0.013 | 0.139 | 0.008 | 0.131 |
| Education | -0.224 | 0.133 | -0.144 | 0.128 |
| *Linguistic Features* |  |  |  |  |
| Provider’s word count | 0.053 | 0.155 | 0.0002 | 0.004 |
| Patient’s word count | 0.038 | 0.157 | 0.033 | 0.147 |
| Patient’s question marks | -0.136 | 0.141 | -0.219 | 0.136 |
| Provider’s affect words | -0.159 | 0.145 | -0.097 | 0.139 |
| Provider’s quantifier | 0.23 | 0.136 | 0.165 | 0.13 |
| *System Use* |  |  |  |  |
| Total clicks | -0.295* | 0.133 | -0.324** | 0.125 |
| Loops | 0.370** | 0.132 | 0.391** | 0.124 |
| *Interaction* |  |  |  |  |
| Loops* Total clicks |  |  | 0.405** | 0.155 |
| Constant | 55.93 | 28.7 | -0.053 | 0.112 |
| **Random effects** |  |  |  |  |
| Between-provider variance | -21.69^***^ | 5.97 | -26.77*** | 6.132 |
| **Log Likelihood** | -1.92E-09 |  | -58.66 |  |
| **Wald Chi2** | 0.118 |  | 30.1 |  |
| *Note: * p < .05. **p < .01. ***p < .001* | |  |  |  |

Table S6. Linear Mixed-effect Model of Linguistic Features and System Use on Patient’s Value Clarity

|  | Model 1 (only main effects) | | Model 2 (interaction effects) | |
| --- | --- | --- | --- | --- |
|  | β | S.E | β | S.E |
| *Patient Demographic* |  |  |  |  |
| Age | 0.056 | 0.132 | 0.077 | 0.124 |
| Education | -0.128 | 0.126 | -0.049 | 0.121 |
| *Linguistic Features* |  |  |  |  |
| Provider’s word count | 0.074 | 0.147 | 0.028 | 0.138 |
| Patient’s word count | -0.216 | 0.149 | -0.222 | 0.139 |
| Patient’s question marks | -0.316* | 0.134 | -0.398** | 0.128 |
| Provider’s affect words | -0.115 | 0.138 | -0.053 | 0.131 |
| Provider’s quantifier | 0.185 | 0.129 | 0.12 | 0.123 |
| *System Use* |  |  |  |  |
| Total clicks | -0.329^*^ | 0.126 | -0.359** | 0.118 |
| Loops | 0.255^*^ | 0.125 | 0.276* | 0.117 |
| *Interaction* |  |  |  |  |
| Loops* Total clicks |  |  | 0.402** | 0.147 |
| Constant | -7.14E-09 | 0.112 | -0.052 | 0.106 |
| **Random effects** |  |  |  |  |
| Between-provider variance | -23.98** | 7.824 | -25.28*** | 6.672 |
| **Log Likelihood** | -59.21 |  | -55.71 |  |
| **Wald Chi2** | 28.35 |  | 40.12 |  |
| *Note: * p < .05. **p < .01. ***p < .001* | | |  |  |

Table S7. Linear Mixed-effect Model of Linguistic Features and System Use on Patient’s Confidence/Certainty about Decision Making

|  | Model 1 (only main effects) | | Model 2 (interaction effects) | |
| --- | --- | --- | --- | --- |
|  | β | S.E | β | S.E |
| *Patient Demographic* |  |  |  |  |
| Age | -0.05 | 0.14 | -0.024 | 0.128 |
| Education | -0.199 | 0.133 | -0.105 | 0.125 |
| *Linguistic Features* |  |  |  |  |
| Provider’s word count | 0.0294 | 0.156 | -0.026 | 0.143 |
| Patient’s word count | -0.109 | 0.158 | -0.113 | 0.143 |
| Patient’s question marks | -0.193 | 0.141 | -0.294* | 0.133 |
| Provider’s affect words | -0.12 | 0.146 | -0.045 | 0.135 |
| Provider’s quantifier | 0.211 | 0.137 | 0.132 | 0.127 |
| *System Use* |  |  |  |  |
| Total clicks | -0.144 | 0.134 | -0.178 | 0.122 |
| Loops | 0.362** | 0.132 | 0.387** | 0.121 |
| *Interaction* |  |  |  |  |
| Loops* Total clicks |  |  | 0.484** | 0.151 |
| Constant | -0.001 | 0.119 | -0.063 | 0.11 |
| **Random effects** |  |  |  |  |
| Between-provider variance | 2.899 | 15.26 | -20.91*** | 5.832 |
| **Log Likelihood** | -61.98 |  | -57.33 |  |
| **Wald Chi2** | 20.13 |  | 34.47 |  |
| *Note: * p < .05. **p < .01. ***p < .001* | |  |  |  |

Figure S2a. Interaction effect of clicks and loops on patient support/advice perception.

Predicted Support Perception

*Note: For illustration purpose, this plot represents the predicted support/advice perception about decision making when 0 and 3 loops are recorded in the CDST.*

*.*

Figure S2b. Interaction effect of clicks and loops on patient value clarity.

Predicted Value Clarity

*Note: For illustration purpose, this plot represents the predicted patient value clarity about decision making when 0 and 3 loops are recorded in the CDST.*

*.*

Figure S2c. Interaction effect of clicks and loops on patient confidence/certainty about decision making.

Predicted Confidence/Certainty

*Note: For illustration purpose, this plot represents the predicted patient confidence/certainty about decision making when 0 and 3 loops are recorded in the CDST.*
